# Metabolic Dysfunction-Associated Steatotic Liver Disease Is Linked to Environmental Sustainability: The Role of the Mediterranean Diet

**DOI:** 10.3390/nu17203206

**Published:** 2025-10-12

**Authors:** Silvia García, Cristina Bouzas, Marina Ródenas-Munar, Violeta Cepeda, Lucía Ugarriza, Miguel Casares, Cristina Gómez, David Mateos, Josep A. Tur

**Affiliations:** 1 Research Group on Community Nutrition and Oxidative Stress, University of the Balearic Islands-IUNICS, 07122 Palma de Mallorca, Spain; 2 Health Research Institute of Balearic Islands (IdISBa), 07120 Palma de Mallorca, Spain; 3 CIBEROBN (Physiopathology of Obesity and Nutrition CB12/03/30038), Institute of Health Carlos III, 28029 Madrid, Spain; 4 Primary Health Care Center Camp Redó, IBSalut, 07010 Palma de Mallorca, Spain; 5 Radiodiagnosis Service, Red Asistencial Juaneda, 07011 Palma de Mallorca, Spain; 6 Clinical Analysis Service, University Hospital Son Espases, 07120 Palma de Mallorca, Spain; 7 Hospital of Manacor, 07500 Manacor, Spain

**Keywords:** Mediterranean diet, intrahepatic fat content, MASLD, environmental impact, sustainability

## Abstract

**Background:** Metabolic dysfunction-associated steatotic liver disease (MASLD) and climate change are major global health challenges. **Aim:** Our aim was to assess the relationship between intrahepatic fat content (IFC) and diet-related environmental impact in a Mediterranean diet (MD)-based intervention. **Design:** The design included a six-month longitudinal analysis within the frame of a FLIPAN randomized controlled trial, including 60 participants aged 40–60 years with MASLD, metabolic syndrome and obesity. **Methods:** IFC expressed as a percentage (%IFC) was measured by magnetic resonance imaging, and dietary intake was assessed via a validated food frequency questionnaire (FFQ). Environmental impacts of diets were estimated using life cycle assessment data from the Agribalyse^®^ database, focusing on greenhouse gas (GHG) emissions, water use, energy use and land use. A composite sustainability score was also calculated. Changes in liver fat and environmental footprints were analyzed using a general linear model (GLM) adjusted for within-subject variability and partial correlation analysis adjusted for energy intake, MD adherence and body weight. **Results:** The participants with the highest %IFC reduction group in the GLM showed the highest decreases in GHG emissions and land use. Water use increased in this same group. Energy use and the composite sustainability score did not differ significantly between groups. Higher %IFC reductions were also associated with higher MD adherence and lower visceral fat. When the adjusted partial correlation analysis for the environmental parameters was performed, only water use remained significant. **Conclusions:** Higher reductions in %IFC were linked to dietary patterns with lower GHG emissions and land use and higher water use. However, when adjusted by energy intake, MD adherence and body weight in continuous modeling, only higher water use was related to lower %IFC. These findings highlight the complexity of achieving environmentally sustainable and health-promoting diets.

## 1. Introduction

Metabolic-associated fatty liver disease (MASLD) has become a leading cause of chronic liver disease worldwide [1]. It is characterized by the presence of more than 5% hepatic steatosis, considering other liver diseases, and includes liver disease due to excessive alcohol consumption [2]. MASLD has been strongly linked to dietary patterns and lifestyle factors, and it is intricately tied to dietary habits characterized by high consumption of ultra-processed foods (UPFs), excessive caloric intake and poor nutritional quality [3,4,5]. These dietary patterns are major contributors to health conditions such as metabolic syndrome (MetS) and visceral fat accumulation, which are also required conditions to assess the definition of MASLD [6]. In contrast, a higher adherence to healthier dietary patterns has been reported as one of the primary treatments for MASLD, since there are no medication treatments widely available. Among those healthier diets, the Mediterranean diet (MD) has been highlighted for leading to a better status of MASLD and helping to have better values of intrahepatic fat content (IFC) [2,3,4].

At the same time, emerging evidence highlights the complex relationship between human dietary patterns and environmental sustainability. Diets rich in animal-based products and UPFs have a high carbon footprint, requiring extensive use of water, land and energy [7,8]. Conversely, diets emphasizing whole foods and promoting the consumption of plant-based options have been aligned with lower environmental impacts and improved health outcomes [9,10]. While previous studies have explored the effects of environmental pollution on visceral fat accumulation and MASLD [11,12,13] or the relationship between MetS, sustainability and dietary patterns [14], the potential for dietary changes to address both MASLD and environmental degradation simultaneously has not been largely investigated.

One interrelated variable between MASLD and the environment could be the MD, as it embodies a dietary model that is both health-promoting and environmentally sustainable [10,15,16,17]. The MD is predominantly plant-based, rich in vegetables, fruits, legumes, whole grains, nuts and olive oil, with moderate consumption of fish and low intake of red and processed meats [18]. This composition aligns with nutritional recommendations for the prevention and management of MASLD due to its anti-inflammatory and antioxidant properties, as well as its ability to reduce insulin resistance and intrahepatic fat accumulation [19,20,21,22]. Simultaneously, the MD has been associated with a lower environmental impact, including reduced greenhouse gas (GHG) emissions, land use and water consumption compared with Western dietary patterns [23]. Therefore, the MD represents a unique opportunity to address both the pathophysiology of MASLD and the environmental burden of food systems through a single, integrative dietary approach.

This research aimed to explore whether promoting sustainable dietary patterns, particularly MD, can contribute to a reduction in liver fat content but also improve diet-related environmental indicators. By exploring how food choices and dietary habits influence both MASLD and environmental sustainability, we aimed to provide a more comprehensive understanding of these interconnected issues. This approach could initiate the way for strategies that target both public health and environmental goals, offering solutions that address both the clinical concerns of MASLD and the broader environmental challenges.

## 2. Methods

### 2.1. Design

This was a six-month longitudinal analysis within the frame of the FLIPAN project, a multicenter, randomized and controlled intervention trial conducted in Spain, registered at ClinicalTrials.gov under identifier NCT04442620 [24]. The FLIPAN study evaluated the effects of a personalized MD combined with physical activity promotion on the prevention and reversal of MASLD in individuals diagnosed with MetS. The intervention integrates tailored nutritional recommendations and lifestyle counseling to improve liver-related outcomes and metabolic health.

### 2.2. Participants, Recruitment and Ethics

Participants were enrolled between June 2018 and January 2020. Eligible participants were aged 40–60 years, had body mass indexes (BMIs) ranging from 27 to 40 kg/m^2^ and met at least three criteria for MetS as defined by the International Diabetes Federation [25]. Of the 143 individuals initially assessed for eligibility, 69 were excluded, 66 due to not fulfilling the inclusion criteria and 3 who declined to participate, resulting in 74 individuals being included in the clinical trial. Of the 74 participants, 14 were excluded due to missing MRI data on the 6-month visit, which did not allow calculation of the change from baseline to 6 months. The final sample therefore consisted of 60 participants. All participants gave written informed consent after receiving a detailed explanation of the study objectives and procedures. The study protocol was approved by the Research Ethics Committee of the Balearic Islands (reference: IB 2251/14 PI) and adhered to the ethical principles of the Declaration of Helsinki.

### 2.3. Sociodemographic Characteristics

Information on participants’ sexes, ages, educational levels (categorized as primary-, secondary- or university-level), employment statuses (active, inactive or retired), alcohol consumption (none, sometimes or regularly) and physical activity levels (none, low, moderate or high) was collected via standardized questionnaires administered at baseline.

### 2.4. Fatty Liver Disease Parameters

Liver fat content was quantified using abdominal magnetic resonance imaging (MRI; Signa Explorer 1.5T, GE Healthcare, Chicago, IL, USA), expressed as a percentage of IFC (%IFC). Hepatic steatosis was further evaluated by ultrasound imaging. Steatosis was graded on a 4-point scale—<5% (grade 0), 5–33% (grade 1), 33–66% (grade 2) and >66% (grade 3)—following established criteria [26]. Liver stiffness and fibrosis were assessed using transient elastography (FibroScan^®^, Echosens, Paris, France) with participants positioned supine and the right arm elevated to optimize access to the intercostal space. Body composition was measured using bioelectrical impedance analysis (Tanita MC780P-MA, Tanita Corp., Tokyo, Japan) to determine visceral fat, in which values ≥13 were considered indicative of elevated risk.

### 2.5. Dietary Parameters

Usual dietary intake was assessed using a validated semi-quantitative food frequency questionnaire (FFQ) comprising 143 items [27,28,29]. This FFQ, administered by trained dietitians, recorded the frequency of consumption of predefined portion sizes across a range of food groups. Frequencies were converted to daily intakes (grams/day) by multiplying the reported frequency by the standard portion size. The food groups analyzed included vegetables, fruits, legumes, cereals, dairy, meats, olive oil, fish, nuts and sweets/pastries. Total daily energy intake (kcal/day) was calculated from those food records using Spanish food composition databases [30,31] by multiplying the intake (g) of each food item by its corresponding energy content and summing across all items. The MD adherence was also reported. Adherence to the MD was evaluated using a 17-item energy-reduced MD adherence questionnaire [32,33]. Each item reflects a component of the MD; adherence was scored as 1 point per compliant item, yielding a total score ranging from 0 to 17, with higher scores indicating greater adherence and dietary quality.

### 2.6. Environmental Parameters

The items included in the administered FFQ were the ones used to estimate the environmental footprint associated with participants’ dietary intake. Life cycle assessment (LCA) data were sourced from the Agribalyse^®^ 3.0.1 database, developed by the French Agency for Ecological Transition (ADEME), in collaboration with the CIQUAL food composition database. This database provides comprehensive environmental impact data for a wide range of food items consumed in the European context, incorporating production and post-farm processes. The LCA methodology adhered to international standards, including ISO 14040 [34] and ISO 14044 [35], and integrated guidelines from LEAP (FAO) and the European Commission’s Product Environmental Footprint (PEF) framework.

Agribalyse^®^ 3.0.1 offers environmental impact data per kilogram of food product, encompassing both agricultural production and downstream processes such as transportation, processing, packaging and retail. Consumer-level factors such as household food waste or transportation from store to home are not included. Additional data from the Ecoinvent^®^ database were used for non-agricultural components such as energy production and transport services.

For the purposes of this study, four environmental indicators were analyzed: GHG emissions, water use, energy demand and land use. GHG emissions were expressed in kilograms of CO_2_ equivalent (kgCO_2_eq), water use in cubic meters (m^3^) and energy use in megajoules (MJ), and land use was assessed through ecological points (Pt), a measure of biodiversity loss based on land occupation and degradation relative to its natural state. For each participant, daily environmental impacts were estimated by multiplying the amount of each food consumed (g) with its environmental value standardized per kilogram of food. These values were summed to obtain the total impact for each environmental indicator per participant and day. Changes in dietary environmental footprints over time were assessed by calculating the difference between baseline and follow-up values, expressed on a per-day basis.

A sustainability score was constructed by integrating four environmental indicators: GHG emissions, energy use, water use and land use. For each participant, the environmental impact of the reported dietary intake was estimated for these indicators, following previously described procedures [15,36]. The median value of each indicator within the sample was used as a cut-off point: values below the median were assigned a score of 1 (lower environmental impact), and those above received a score of 0 (higher environmental impact). The four partial scores were summed to obtain a total sustainability score ranging from 0 to 4, where higher values indicated a more environmentally sustainable diet. Changes in this score between baseline and 6 months were also evaluated.

### 2.7. Statistics

Statistical analyses were performed using SPSS software (version 27.0; IBM Corp., Armonk, NY, USA). Continuous variables were expressed as means and standard deviations, while categorical variables were expressed as frequencies and percentages. Group comparisons were carried out using the Chi-square test for categorical variables and Student’s *t*-test for continuous variables. Changes in %IFC from baseline to 6 months were separated into two groups: G1 represented a higher reduction in %IFC (≤−3.2%) and G2 represented a lower reduction or increase in %IFC (˃−3.1). This can be seen in Table 1. Additionally, Table 1 also shows the sociodemographic analysis separating the total sample into two different groups, completers and dropouts, to assess potential missing data bias. The general linear model (GLM) for repeated measures was used to assess the relationships between changes in %IFC, environmental indicators, MD adherence and fatty liver disease parameters other than IFC over the 6-month period. The GLM was adjusted for within-subject variability, aiming to account for individual-level differences across time points. Bonferroni’s post-hoc test was applied to identify statistically significant differences (*p* < 0.05) between groups within times (a), between times within groups (*) and between time*group interactions. Table 2 shows the GLM analysis. Effect estimates (B coefficients), 95% confidence intervals and partial eta-squared (η^2^ partial) values have also been calculated in Table 2 to provide information on the magnitude and precision of the observed effects. Finally, a partial correlation analysis was carried out between %IFC and environmental parameters to see these relations using continuous modeling. This partial correlation analysis was adjusted for changes in energy intake, MD adherence and body weight. These analyses can be seen in Table 3.

## 3. Results

Table 1 shows the sociodemographic characteristics of the study sample distributed into two groups according to changes in the IFC (%) during a 6-month period. The variables determined were age, sex, educational level, job situation, alcohol consumption and physical activity. None of them presented significant differences between groups, reflecting a homogeneity in sociodemographic characteristics across the groups created based on our grouping variable (%IFC change), which allows us to avoid potential confounding factors.

A total of 60 participants completed this study, while 83 participants dropped out. Table 1 also shows the comparison between completers and dropouts. No significant differences were observed in age, educational level, job situation, alcohol consumption or physical activity (*p*-value > 0.05), suggesting that the completers were largely similar to the dropouts for these characteristics. However, a higher proportion of men (63.3% vs. 44.6%) and a lower proportion of women (36.7% vs. 55.4%) were present among the completers compared to dropouts (*p*-value = 0.03).

Table 2 shows the diet-related environmental indicators: GHG emissions (kg CO_2_eq), water use (m^3^), energy use (MJ) and land use (Pt), along with the environmental sustainability score, adherence to the MD, elastography values (kPa), hepatic steatosis level and visceral fat, according to 6-month changes in %IFC. GHG emissions, water use, land use, MD adherence and visceral fat were significantly associated with changes in %IFC (*p*-values < 0.05 for time*group interactions).

The participants with higher reductions in %IFC (G1) showed a significant decrease in GHG emissions, which dropped from 5.3 ± 1.7 to 4.7 ± 1.4 kg CO_2_eq (Δ = −0.5 ± 1.5), whereas emissions remained virtually unchanged in the group with lower reductions or increased %IFC (G2), moving from 5.4 ± 1.6 to 5.4 ± 1.9 kg CO_2_eq (Δ = +0.04 ± 1.1). Land use followed a similar pattern, with a substantial reduction observed in G1 (from 276.1 ± 98.1 to 236.3 ± 90.1 Pt; Δ = −39.8 ± 81.1), while G2 only experienced a minor decrease (from 266.2 ± 86.9 to 261.8 ± 111.1 Pt; Δ = −4.3 ± 63.4). Conversely, water use increased in G1 (from 10.1 ± 2.8 to 11.6 ± 4.4 m^3^; Δ = +1.5 ± 3.8), while it decreased slightly in G2 (from 12.3 ± 4.8 to 11.5 ± 4.5 m^3^; Δ = −0.7 ± 3.4).

Adherence to the MD was strongly associated with %IFC changes. Both groups improved their MD adherence, but the increase was significantly greater in G1. Specifically, G1 improved from 7.9 ± 2.5 to 12.8 ± 2.6 points (Δ = +4.8 ± 3.1), nearly doubling the increase observed in G2, which went from 8.1 ± 2.9 to 10.9 ± 2.5 points (Δ = +2.7 ± 2.8).

Visceral fat content followed a similar trend. Both groups experienced reductions, but the changes were more pronounced in G1. Visceral fat decreased from 14.1 ± 3.1 to 12.9 ± 2.8 points in G1 (Δ = −1.3 ± 1.1), while in G2, the decrease was minimal, from 13.6 ± 3.9 to 13.4 ± 3.6 (Δ = −0.2 ± 1.1).

The other variables, including energy use, environmental sustainability score, elastography results and hepatic steatosis level, did not show significant associations between groups. Additionally, Table 2 includes effect estimates (B coefficients), 95% confidence intervals and partial eta-squared (η^2^ partial) values.

Table 3 presents the partial correlations between 6-month changes in environmental indicators and %IFC, adjusted for changes in total energy intake, MD adherence and body weight. A significant inverse correlation was observed between changes in water use and %IFC (r = −0.301, *p* = 0.02), indicating that greater reductions in %IFC were associated with increased water use in the production of the food consumed. No significant associations were found between %IFC changes and the remaining environmental indicators, including GHG emissions, energy use, land use and sustainability score (*p*-values > 0.05).

## 4. Discussion

The present study reinforces the interconnection between health, diet and environmental sustainability. Greater reductions in %IFC after six months of adherence to the MD were associated with lower dietary environmental impacts, particularly in terms of GHG emissions and land use, alongside an increase in water use. However, when total energy intake, MD adherence and body weight were considered, these reductions were attenuated and only the increase in water use remained statistically significant.

GHG emissions, water use, energy use and land use are the four most common environmental parameters used in the literature to assess the environmental impact that comes with a diet [16,37,38,39,40,41]. Among them, GHG emissions are the most relevant since they are the main drivers of the greenhouse effect and global warming and are the ones tackled to reduce global environmental impact [42,43,44]. The GLM analysis shows how participants with higher reductions in their %IFC were having more than tenfold reductions of dietary GHG emissions compared with the group with lower reductions in %IFC. This suggests a potential dual benefit where improvements in liver fat content may be associated with diets that also generate lower environmental pressure. These initial results reinforce the notion that the MD is a dietary pattern capable of improving both environmental sustainability and health parameters, as the environmental impact calculated reflects the production of the foods actually consumed by the participants. The current findings also show a reduction in land use among participants with higher decreases in %IFC. Again, this reduction in environmental impact appears to parallel the metabolic improvements observed. To the best of our knowledge, no previous studies have specifically examined the association between %IFC and environmental impact indicators such as GHG emissions or land use, which adds novelty and relevance to the present findings. Some previous studies have explored the relationship to dietary environmental impact, particularly GHG emissions and other health-related outcomes, such as MetS, body composition or the risk of non-communicable diseases [14,45,46], suggesting a convergence between health benefits and environmentally sustainable eating patterns. Other studies reported the effect of the MD on environmental impact, showing a general trend of lower GHG emissions and land use when eating diets rich in plant foods compared with those rich in animal products, which is one of the MD characteristics [47,48,49].

However, when continuous modeling and further adjustments were made for total energy intake, adherence to the MD and body weight, the associations between %IFC and both GHG emissions and land use lost statistical significance. This attenuation suggests that part of the relationship between %IFC and these environmental indicators may be explained by differences in overall energy consumption or dietary composition rather than by an independent effect of liver fat changes, meaning that individuals achieving greater improvements in liver fat content may also be those adhering more closely to the MD or reducing their energy intake: factors that could be linked to lower GHG emissions and land use.

Regarding water use, the current results showed an increase in water use in participants with higher reductions in %IFC. While animal-based products, especially red meat, remain the most water-intensive overall [50,51,52], the replacement of low-nutrient UPFs with minimally processed, nutrient-dense options may still lead to a higher water footprint, particularly in the case of nuts or fresh plant-based foods [7,53]. After adjusting the analyses for total energy intake, MD adherence and body weight, water use remained the only environmental indicator significantly associated with %IFC. These findings highlight the complexity of designing dietary patterns that optimize both health and environmental outcomes.

Although we observed reductions in energy use among the participants with the highest decreases in %IFC, these differences were not significant. Therefore, we decided to calculate a sustainability score [15], which integrates the four environmental indicators calculated in the current study, to provide a more comprehensive perspective on environmental impact. The current results show that this composite sustainability score was not significantly associated with changes in %IFC among our participants. This finding further highlights the complexity of evaluating environmental impact, which is influenced by numerous interrelated factors [54]. Both energy use and the overall sustainability score showed no significant associations with %IFC either in the GLM analysis or after adjusting for energy intake, MD adherence and body weight in the partial correlation analysis. While analyzing each indicator separately allows us to understand their individual contributions, it also demonstrates the importance of a holistic approach, as focusing solely on the aggregated score may obscure potentially meaningful individual effects, particularly in intervention settings where multiple dietary and metabolic factors interact.

The current findings confirmed that adherence to the MD and reductions in visceral fat were strongly correlated with decreases in %IFC. Although this association is well-established in the literature and is not a novel result [55,56,57], it reinforces the intrinsic connection between dietary quality and improvements in both metabolic health and environmental outcomes. As mentioned, the MD is characterized by a high presence of plant-based foods, healthy fats and minimally processed ingredients and has consistently been associated with reduced risk of suffering from non-communicable diseases and lower environmental impact [17,58]. This dual benefit highlights the importance of promoting dietary patterns that are not only effective for the prevention and management of metabolic disorders, such as MASLD, but also aligned with environmental sustainability goals. Ultimately, the current findings emphasize that meaningful changes in individual health metrics, such as %IFC, can be achieved through dietary shifts that simultaneously contribute to broader ecological benefits, supporting the integration of health and sustainability objectives in public health nutrition strategies.

From a practical perspective, these findings provide several key considerations for developing “pro-liver and pro-planet” dietary strategies within the MD framework. Such an approach should prioritize plant-based and minimally processed foods, including fruits, vegetables, legumes, whole grains and nuts, while using virgin or extra-virgin olive oil as the main source of fat and moderating the intake of red and processed meats and UPFs [59]. However, these recommendations must be implemented with a balanced perspective. As observed in our study, following a more plant-based diet, such as the MD, and increasing the consumption of fresh foods may lead to a higher water footprint, even when adjusting for energy intake, MD adherence and body weight.

Of those three variables, the overall energy intake of the diet remains a critical factor. Although the GLM did not show statistically significant associations, the observed reduction in energy intake in G1 may partially explain some of the observed environmental and health trends [14,46,60]. A sustainable dietary pattern may negatively affect both environmental impact and body composition if caloric intake exceeds individual needs. Therefore, designing truly “pro-liver and pro-planet” menus requires optimizing both the nutritional and environmental dimensions of the diet to achieve metabolic health and sustainability goals simultaneously.

### Strengths and Limitations of This Study

This study benefits from several notable strengths. First, the 6-month longitudinal follow-up provides a robust framework for assessing changes in %IFC and dietary environmental impact over time, allowing for the observation of meaningful metabolic and behavioral changes. Another strength lies in the recording of the dietary intake, which was performed by trained dietitians using validated questionnaires. A first sociodemographic analysis was carried out to assess if those variables could be confounding factors, but none of them was significantly associated with changes in %IFC. However, the GLM analysis was adjusted for within-subject variability, and the partial correlation analysis was adjusted by energy intake, MD adherence and body weight to avoid further bias. The inclusion of four major environmental indicators, GHG emissions, water use, land use and energy use, allowed for a multidimensional evaluation of diet sustainability, which is rarely addressed in the context of clinical interventions targeting metabolic liver diseases.

This study also has limitations. Although the data were derived from an RCT, the analyses of environmental outcomes were observational and exploratory. As such, while the longitudinal design supports temporal relationships, causal inference remains limited. Even though it is a neighboring country, using the French Agribalyse database may have introduced minor differences due to country-specific food composition and production practices in Spain. The participants were 40 to 60 years old, which means that the data cannot be extrapolated for younger adults or children.

## 5. Conclusions

This study adds to the growing body of evidence supporting the integration of metabolic health and environmental sustainability. Greater reductions in %IFC after six months of adherence to the MD were initially associated with lower dietary GHG emissions and land use alongside an increase in water use. However, after using continuous modeling and accounting for total energy intake, MD adherence and body weight, only the association with water use remained significant. These findings suggest that improvements in liver fat may parallel reductions in environmental impact, although part of this relationship appears to be explained by differences in energy intake and dietary composition. The MD, characterized by its predominance of plant-based and minimally processed foods, remains a promising framework for promoting both metabolic and environmental benefits. Nevertheless, the observed increase in water use highlights the complexity of achieving truly holistic sustainability through diet. Future research should further investigate these relationships in larger and more diverse populations using refined sustainability metrics that capture both health and environmental dimensions in an integrated manner.

## Figures and Tables

**Table 1:** Sociodemographic characteristics of the sample are at baseline according to changes in %IFC within 6 months.

	%IFC Higher Reduction (§) *n* = 30	%IFC Lower Reduction or Increase (§) *n* = 30	*p*-Value
Age	Mean (SD)		
	52.3 (6.8)	53.3 (6.6)	0.56
Sex	*n* (%)		
Men	20 (66.7)	18 (60)	0.59
Women	10 (33.3)	12 (40)	
Educational Level	*n* (%)		
Primary	9 (30)	12 (40)	0.65
Secondary	13 (43.4)	12 (40)	
University	8 (26.7)	6 (20)	
Job Situation	*n* (%)		
Not working	4 (13.3)	6 (19.9)	0.70
Working	24 (80)	20 (66.7)	
Retired	2 (6.7)	4 (13.3)	
Alcohol Consumption	*n* (%)		
None	12 (40)	7 (23.3)	0.38
Yes, sometimes	14 (46.7)	18 (60)	
Yes, regularly	4 (13.3)	5 (16.7)	
Physical Activity	*n* (%)		
None	14 (46.7)	15 (50)	0.86
Low	11 (36.7)	12 (40)	
Moderate	4 (13.3)	2 (6.7)	
High	1 (3.3)	1 (3.3)	
	**Completers** ***n*** **= 60**	**Dropouts** ***n*** **= 83**	***p*** **-Value**
Age	Mean (SD)		
	52.8 (6.7)	51.8 (7.3)	0.49
Sex	*n* (%)		
Men	38 (63.3)	37 (44.6)	0.03
Women	22 (36.7)	46 (55.4)	
Educational Level	*n* (%)		
Primary	21 (34.0)	33 (39.7)	0.81
Secondary	25 (41.7)	29 (35.0)	
University	14 (23.3)	21 (25.3)	
Job Situation	*n* (%)		
Not-working	8 (13.4)	11 (13.2)	0.78
Working	46 (76.6)	67 (80.7)	
Retired	6 (10.0)	5 (6.0)	
Alcohol Consumption	*n* (%)		
None	19 (31.7)	35 (42.2)	0.51
Yes, sometimes	32 (53.3)	35 (42.2)	
Yes, regularly	9 (15.0)	13 (15.7)	
Physical Activity	*n* (%)		
None	29 (48.3)	41 (49.4)	0.62
Low	23 (38.3)	26 (31.3)	
Moderate	6 (10.0)	14 (16.9)	
High	2 (3.3)	2 (2.4)	

Abbreviations: IFC: intrahepatic fat content; SD: standard deviation; §: differences in %IFC between baseline and 6-month follow-up calculated with magnetic resonance imaging (MRI) and distributed in two groups. Differences in means between groups were tested using Student’s *t*-test. Differences in prevalences across groups were examined using χ^2^.

**Table 2:** Diet-related environmental indicators according to 6-month changes in IFC (%).

		%IFC Higher Reduction (§), *n* = 30	%IFC Lower Reduction or Increase (§), *n* = 30	B Coefficient	95% Confidence Intervals	η^2^ Partial	Time*Group ^‡^
		Mean (SD)					
GHG Emissions (kg CO_2_eq)	Baseline	5.3 (1.7)	5.4 (1.6)	14.2	−4.8–33.3	0.07	0.04
	6 months	4.7 (1.4)	5.4 (1.9)				
	▲	−0.5 (1.5) *	0.04 (1.1)				
Water Use (m^3^)	Baseline	10.1 (2.8)	12.3 (4.8)	0.2	−52.1–52.5	0.08	0.02
	6 months	11.6 (4.4)	11.5 (4.5)				
	▲	1.5 (3.8) *	−0.7 (3.4)				
Energy Use (MJ)	Baseline	62.2 (20.2)	65.8 (20.9)	123.2	−105.1–351.5	0.04	0.16
	6 months	57.9 (16.7)	66.4 (21.8)				
	▲	−4.3 (17.5)	0.5 (14.1)				
Land Use (Pt)	Baseline	276.1 (98.1)	266.2 (86.9)	947.5	−74.8–1969.9	0.08	0.02
	6 months	236.3 (90.1)	261.8 (111.1)				
	▲	−39.8 (81.1) *	−4.3 (63.4)				
SScore (points)	Baseline	2.1 (1.4)	1.8 (1.6)	−4.3	−27.7–19.1	0.003	0.68
	6 months	2.2 (1.6)	1.7 (1.7)				
	▲	0.1 (1.8)	−0.03 (1.3)				
MD-Adh (Points)	Baseline	7.9 (2.5)	8.1 (2.9)	−20.1	−62.4–22.1	0.13	<0.01
	6 months	12.8 (2.6) ^a^	10.9 (2.5) ^a^				
	▲	4.8 (3.1) *	2.7 (2.8) *				
Energy Intake	Baseline	2332.6 (688.4)	2261.9 (552.9)	813.3	−8563.8–10,190.5	0.03	0.18
	6 months	2065.7 (423.2)	2221.3 (750.3)				
	▲	−266.8 (722.2) *	−40.5 (562.1)				
Elastography (Kilopascals)	Baseline	5.3 (1.5) ^a^	4.7 (1.3) ^a^	1.5	−27.4–30.5	0.08	0.07
	6 months	5.4 (1.6)	5.2 (1.5)				
	▲	−0.1	0.8 (1.6) *				
Hepatic Steatosis level	Baseline	2.0 (0.2) ^a^	1.6 (0.4) ^a^	−4.5	−19.5–10.4	0.07	0.09
	6 months	1.3 (0.7)	1.6 (0.7)				
	▲	−0.6 (0.8) *	−0.1 (0.7)				
Visceral Fat (points)	Baseline	14.1 (3.1)	13.6 (3.9)	−1.6	−18.6–15.5	0.15	<0.01
	6 months	12.9 (2.8)	13.4 (3.6)				
	▲	−1.3 (1.1) *	−0.2 (1.1)				

Abbreviations: IFC: intrahepatic fat content; SD: standard deviation; GHG: greenhouse gas; SScore: sustainability score (integrating the four environmental parameters); MD-Adh: Mediterranean diet adherence; §: differences in %IFC between baseline and 6-month follow-up calculated with magnetic resonance imaging (MRI) and distributed in two groups; ▲: change between baseline and 6 months. The GLM was adjusted for within-subject variability. Bonferroni’s post-hoc test was applied to identify statistically significant differences (*p* < 0.05) between groups within times (^a^), between times within groups (*) and between time*group interactions. Effect estimates (B coefficient), 95% confidence intervals and partial eta-squared (η^2^ partial) values were calculated. ^‡^ Bonferroni’s post-hoc test time*group interactions.

**Table 3:** Partial correlation analysis of 6-month changes in environmental indicators and IFC (%).

	%IFC Changes	
	R (Correlation Coefficient)	*p*-Value
GHG Emissions (kg CO_2_eq) change	−0.005	0.96
Water Use (m^3^) Change	−0.301	0.02
Energy Use (MJ) Change	−0.082	0.54
Land Use (Pt) Change	0.042	0.75
SScore Change	1.000	0.16

Abbreviations: IFC: intrahepatic fat content; GHG: greenhouse gas; SScore: sustainability score (integrating the four environmental parameters). Partial correlation analysis was adjusted for changes in energy intake, MD adherence and body weight.

## Data Availability

There are restrictions on the availability of the data for this trial due to the signed consent agreements around data sharing, which only allow access to external researchers for studies following project purposes. Requestors wishing to access the trial data used in this study can make a request to pep.tur@uib.es.
